# Using government documents in open pedagogy: A method for engagement and building skills

**DOI:** 10.1016/j.mex.2021.101612

**Published:** 2021-12-23

**Authors:** Stephanie Hallam, Pat Willingham

**Affiliations:** Southeast Missouri State University, United States of America

**Keywords:** Open educational resource, U.S. government documents, Information literacy, Open pedagogy

## Abstract

This method utilizes a template for an open pedagogy lesson plan using US government documents that can be applied to academic levels ranging from elementary to post-secondary. This method addresses concerns about OER quality, exposes students to the wealth of information in U.S. government documents, and develops research skills. The topic can be changed to accommodate diverse subject areas, and the duration can be adjusted to meet the instructional expectations. Students gain familiarity with government document search engines and resources which they can use throughout their lives. Through this process they strengthen their critical thinking, evaluative, and analytical skills as they explore a set of documents to create a narrative based on an overarching theme. Students use a worksheet to guide their narrative development. Examples of this method, as applied to a history topic, are included.

An existing open pedagogy method to increase student engagement was used (Hollister, 2020). The proposed application of this method results in:

• A flexible lesson that introduces students to various types of U.S. government documents.

• An approach to develop research skills while generating a project that can vary based on discipline and academic level.

## Graphic abstract

**Figure:**
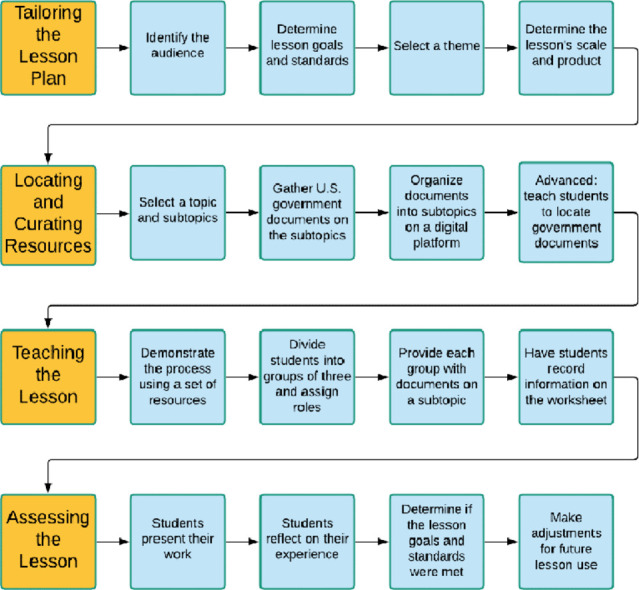

Specifications table

**Table:** 

Subject Area/ psychology:	Psychology
More specific subject area:	Open pedagogy, U.S. government documents, Open educational resources
Method name:	Open Pedagogy
Name and reference of original method:	Hollister, C. (2020). Using Open Pedagogy to Engage LIS Students: A Case Study. *Journal of Librarianship & Scholarly Communications*, 8, 1-17. https://doi.org/org/10.7710/2162-3309.2357
Resource availability:	Link to research guide: https://semo.libguides.com/c.php?g=930009 Link to presentation: https://docs.google.com/presentation/d/1MyUmFtyVAwFIBNn8yMXu2MJRfu1zxiAP83LoR5HknkQ/edit?usp=sharing Link to worksheet: https://docs.google.com/document/d/1dupLSAy4SriW-RvmBpUBdH_P_CzNEDqot9BbQmu0XXs/edit?usp=sharing

## Introduction

Open pedagogy is a teaching method that can deepen student engagement through active learning as learners create knowledge. This idea developed into OER-enabled pedagogy which combines OER and open pedagogy [2]. OER-enabled pedagogy allows learners to create OER, shifting from passive acquisition of information to active knowledge production through the synthesis of resources [3,4]. There are two key facets to the process: OER expands access to knowledge while open pedagogy expands access to knowledge creation [5]. Through active learning and higher order critical thinking skills, student engagement increases [6]. As a result, research becomes a puzzle to be solved and assignments can solve real-life problems. Those solutions can be shared as OER.

The term “disposable assignments” has been used to describe traditional essays, term papers, and other assignments that have no life or purpose beyond meeting a requirement for a particular course [7]. Instead, these exercises should be replaced with “renewable assignments” which can be structured to contribute to the body of knowledge openly available. This addresses the concern that OER are often disconnected from real life and become ineffectual when taken out of instructional contexts [8]. Building upon the idea of renewable assignments, a series of scaffolded assignments was created for psychology students which involved using Wikipedia to engage in activities such as adding citations, checking links, or, for higher-level students, even writing new articles [9]. All these activities were designed to educate students and improve the quality of information available to the public. Another assignment brought this full circle by having each student in a graduate course write a chapter for their course textbook, which was then shared as an OER under a Creative Commons license [1]. Each of these instances addressed real-life problems while contributing to the OER corpus, benefiting students, and expanding knowledge for those beyond the classroom.

Despite these demonstrated benefits, concern about quality has been a barrier to using open pedagogy. For example, OER do not require a standard evaluation process such as one used by publishers [3]. The concern for quality resources is addressed in this method by using United States of America federal government documents, which are freely available to the public and give students and faculty access to information across many disciplines.

A barrier to using government documents is the lack of awareness by librarians, faculty, and students [10]. Contributing to this barrier is the fact that many librarians are not comfortable using government documents and avoid teaching about them [11]. However, a review of undergraduates’ annotated bibliographies revealed that, when required to include two websites, government resources were cited third after books and journals [12]. Clearly, government resources would be used more frequently if students were taught about their credibility and relevance. Through government documents, students can be introduced to new sources of authoritative information while improving their information evaluation skills and the quality of their internet research [13]. Use of these resources encourages learners to develop the lifelong skills of locating, evaluating, and using information in sources that can be accessed throughout their lives [14]. Open pedagogy also addresses the concern of quality by providing learners with a contextual framework to evaluate resources for their research. Context is imperative in assessing a resource's quality and educational benefit [2].

Despite the potential benefits, using U.S. government documents in open pedagogy has gained little traction with librarians and educators. This may be due to the lack of application methodology. The proposed methodology could encourage use of government documents in a way that greatly benefits students and instructors.

## Method details

### Tailoring the lesson plan

Based on the audience and academic level, determine the lesson's goals and standards to be met. This lesson plan can have learning goals that align with many national standards and at multiple educational levels. Align the lesson to the standards for the specific academic setting.

For example, this lesson's outcomes could be aligned with the Association of College & Research Libraries’ (ACRL) Frameworks [15]. Specifically, this method can connect to either or both of the frames below:

• “Information Creation as Process: Information in any format is produced to convey a message and is shared via a selected delivery method. The iterative processes of researching, creating, revising, and disseminating information vary, and the resulting product reflects these differences.”

 The goals of the lesson can be for students to evaluate a collection of resources to select information, analyze it, and synthesize it into a narrative. By using these goals with this method, students will participate in the information creation process. They will experience researching, creating, and revising as they develop a narrative using government resources. This nonlinear process will challenge students to deepen their research as questions and themes develop.

• “Scholarship as Conversation: Communities of scholars, researchers, or professionals engage in sustained discourse with new insights and discoveries occurring over time as a result of varied perspectives and interpretations.”

 Based on the above goals, this method will encourage students to create “renewable” assignments as they develop insights and discoveries through their research. The created artifacts can be shared and contributed to scholarly conversation.

The lesson's outcomes likewise can be aligned to state K-12 or higher education standards across disciplines.

To help students create a narrative with the government documents, the instructor should select a broad theme. The theme will guide the selection and interpretation of these materials and focus the research. For example, the theme of sacrifice, used in the original research article, was applied to the topic of World War II heroes (e.g., subtopics included Julia Child, Jackie Robinson, and Glenn Miller). Students could use the theme as a lens to view resources. This supports inquiry-based learning and helps learners create narratives out of the information while reducing the potential for confusion. Without this direction, students might struggle to find connections, see value in the artifacts, or look for things that interest them. After selecting a theme, the instructor should determine the lesson's scale and product. The activity can be scaled to reflect the desired artifacts which could range from a simple oral presentation to a semester-long research project.

The lack of an instructor's scope and depth of knowledge on the topic or the resources can pose a challenge when selecting themes or topics. The instructor's knowledge is essential when determining the appropriateness of a theme or topic for the lesson's goals and students’ academic level.

### Locating and curating lesson resources

Select a topic for searching and identify six to seven subtopics to assign to groups within the class. Under title 17 section 105 of the United States Code, most documents created by federal agencies fall under public domain and therefore qualify as OER [16]. The United States government publishes documents, increasingly born digital, on a variety of topics. USA.gov is recommended, as noted in the original research article, as an entry-level search portal because of its ease of use, while a librarian can provide support locating other resources.

Locate 3-5 U.S. government resources for each subtopic and organize these resources on a website. This could be any platform to organize and share the resources. For example, https://semo.libguides.com/c.php?g=930009, included in the original research article, provides lists of topics and subtopics. Instructors can utilize a range of government agency instructional resources such as the Library of Congress or the Central Intelligence Agency to locate curated collections. Examples are listed under “Instructional Resources” in the site linked above. Advanced students could be taught to use government websites and portals to locate their own materials. Additional examples and methods for locating and curating lesson resources are available in the original companion paper.

Determining the best resources to engage learners and provide access can be a challenge. Some government resources are print materials which include eye-catching images that quickly grab attention. However, print resources do not provide practical access for multiple users. To address this challenge, print resources can be used in the model narrative but electronic resources should be prioritized for learners to increase access, searchability, and distribution of this method and lesson plan.

### Teaching the lesson

**Fig. 1:**
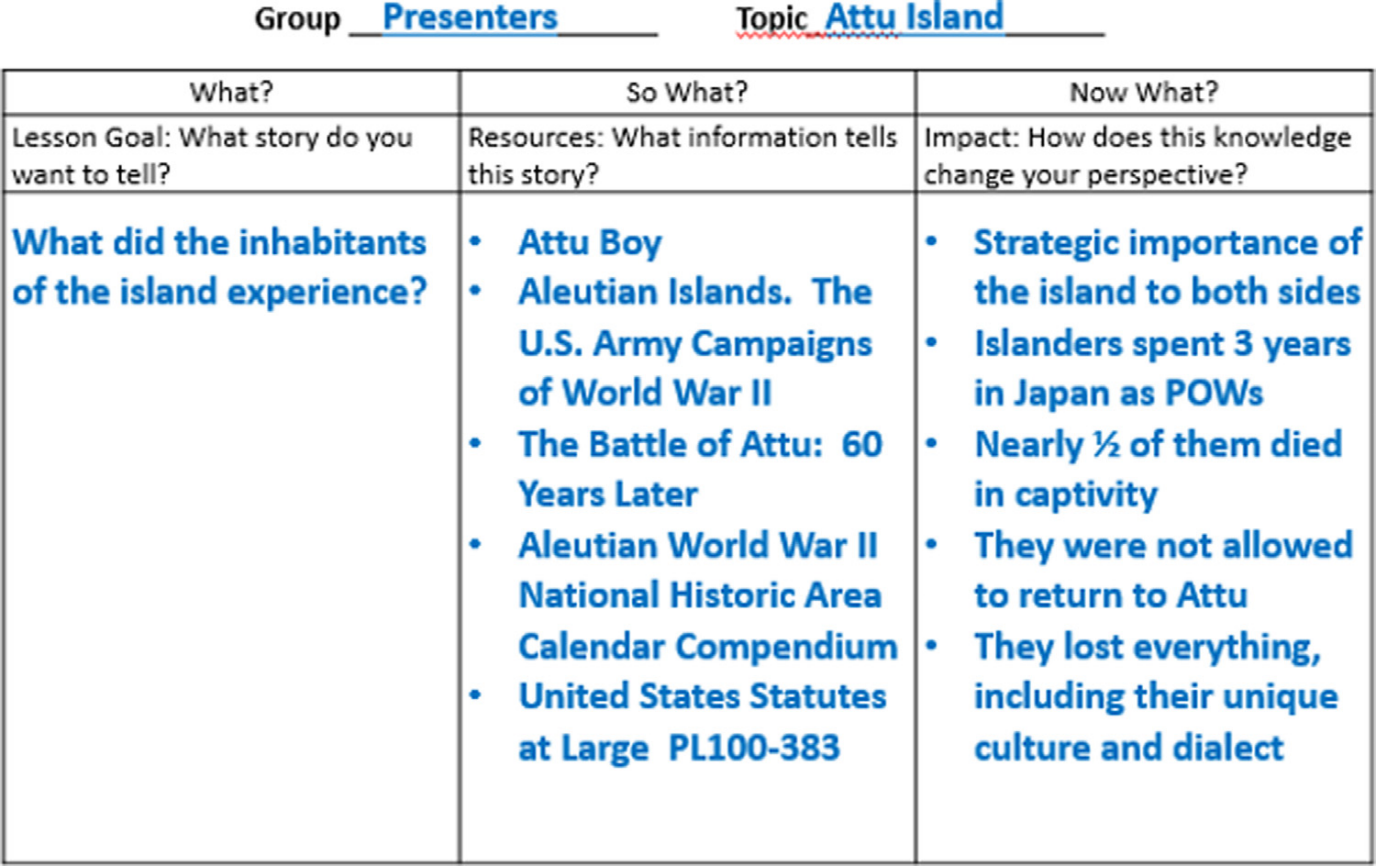
Sample completed worksheet.

Introduce students to the lesson by walking through a sample project using the theme and a set of resources that will not be used by students. Demonstrate using the worksheet (Fig. 1). Share examples of others’ works that have used the same open pedagogy process. These examples could include prior student work or published documents such as *Attu Boy* [17] or *When the Akimotos Went to War* [18]. Include a brief narrative that models analyzing, evaluating, and integrating resources. For example, the original research article used five resources on Attu Island, found in the research guide linked above, to answer the questions on the worksheet. Based on these answers, a narrative was created that provided a synopsis of the experience and sacrifice of the Aleutian residents. Resources such as maps and images were shared to tell the story. Then the presenters reflected on how this information reshaped their perspective. After sharing the narrative, the presenters articulated the steps taken to analyze, evaluate, and integrate the resources.

**Fig. 2:**
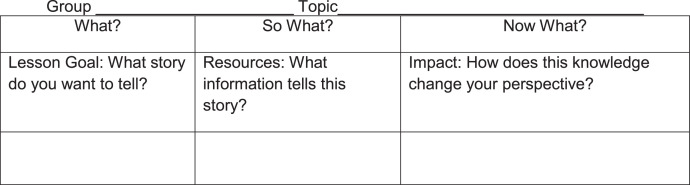
Worksheet sample.

Divide students into groups of three and assign each member a role: recorder, manager, or presenter. These can be adjusted and defined to meet the academic level and intended product. For example, the recorder could use the worksheet to record the group's work, the manager could keep the group focused and ensure that the task is being completed, and the presenter could lead how the knowledge will be communicated. This will encourage teamwork and divide labor so the project can be completed during the allotted time. Assign or allow group-selection of topics (multiple groups presenting on the same materials can result in vastly divergent presentations and new insights). Provide students a worksheet to guide and record their work (Fig. 2).

### Assessing the lesson

At the conclusion of the project, the student groups will share their knowledge as a new work. Depending upon the format of the selected lesson product, it can be shared either with the entire class or directly to the instructor. After sharing their work, have students reflect on the process and the product. Encourage them to consider how they analyzed, evaluated, and integrated information. The reflection formats can vary based on the audience, goals, standards, and project. It could also occur as a simple discussion or a more formal written document.

After students have shared their product and reflections, evaluate the lesson from an instructor's perspective to determine if the lesson goals and standards were met. Focus the evaluations on the process rather than the product. Based on these evaluations, determine if changes need to be made before future applications of this method. Make any necessary changes to improve the use of this method with future students. These improvements could focus on changing the resources, adjusting the product, or changing how the product is presented.

## Conclusion

Implementing this method has produced a range of products. The authors have presented this approach seven times and it was warmly received by instructors and librarians at all academic levels and in diverse subject areas. It is easy to use and highly adaptable. It engages students and fosters critical thinking. And the ready availability of public domain government resources removes the barriers of access and cost. Furthermore, teaching students to locate and use government resources provides them with a mechanism for finding information on virtually any topic, thereby strengthening their skills as lifelong learners.

## Declaration of Competing Interest

The authors declare that they have no known competing financial interests or personal relationships that could have appeared to influence the work reported in this paper.
